# Crosstalk between stromal, immune, and ovarian cancer cells in lipid-rich tumor microenvironment exhibits proliferative features

**DOI:** 10.3389/fimmu.2025.1614815

**Published:** 2025-07-10

**Authors:** Qinsiyu Ma, Rui Kang, Ruiyue Xu, Yifu Guan, Shijie Chang, Shuo Li

**Affiliations:** ^1^ Department of Biochemistry and Molecular Biology, School of Life Sciences, China Medical University, Shenyang, Liaoning, China; ^2^ Department of Biomedical Engineering, China Medical University, Shenyang, Liaoning, China

**Keywords:** lipid metabolism, ovarian cancer, stromal tumor microenvironment, immune response, immunosuppresive TME

## Abstract

Lipid metabolism reprogramming has long been noticed as the hallmark of ovarian cancer, in order to maintain proliferative features including rapid cell division, metastasis capability, and chemotherapy resistance, as well as to survive under environmental stress, alteration of lipid metabolic pathways takes place, especially over-expression of rate-limiting enzymes, enhances lipid uptake, fatty acid synthesis, β-oxidation, lipid storage, and cellular membrane construction. In lipid-rich ascites and omental tumor microenvironments, the biological functions of stromal and immune cells change, forming a premetastatic niche and immunosuppressive tumor microenvironment via modifying extracellular matrix components and secreting cytokines. The crosstalk between stromal, immune, and ovarian cancer cells results in tumor proliferation, metastasis, and escape of immune surveillance. Given the importance of lipid metabolism for ovarian cancer survival, targeting lipid metabolism key enzymes in ovarian cancer or stromal tumor microenvironment may bring novel insights for ovarian cancer treatment.

## 1 Introduction

Ovarian cancer is one of the most lethal gynecological malignancies. As the eighth most common cancer among women worldwide, ovarian cancer accounts for an estimated 3.7% of cases and 4.7% of cancer deaths in 2020 (1). The current primary treatment for ovarian cancer still consists of cytoreductive surgery and chemotherapy that combines platinum compounds and taxanes. Currently, acquired drug resistance in ovarian cancer is mainly considered the result of drug efflux caused by P-glycoprotein, encoded by ATP-binding cassette B1 (ABCB1), which is significantly affected by various lipid compounds especially those residing in their close proximity in the plasma membrane. However, the development of P-glycoprotein as a therapeutic target has been unsuccessful (2). Emerging evidence implicates a key role for non-mutational drug resistance mechanisms underlying the survival of residual cancer “persister” cells. Drug-tolerant persister cancer cells exhibited phenotypic plasticity, including metabolic adaption, stem-like characteristics, and dormancy to temporarily survive therapeutic pressure (3). Hence, there is an urgent demand for new clinical biomarkers targeting chemotherapy resistance occurrence and exploring novel therapeutic strategies.

Ovarian cancer features a unique tumor microenvironment (TME) characterized by hypoxia, large amounts of fat in the omentum, and ascites, all of which contribute to complex biological behaviors such as cellular metabolic reprogramming, reduced perfusion of chemotherapeutic drugs, and the promotion of an immunosuppressive environment. In order to survive in this environment, tumor cells undergo reprogramming of lipid metabolism to adapt to the metabolic and oxidative stress in the microenvironment, which mostly involves dysregulation of metabolic key enzyme activities and expression. During metabolic stress conditions, fatty acids (FAs) stored in lipid droplets (LDs) are hydrolyzed via activating fatty acid oxidation (FAO). This process aims to maintain rapid cancer cell division via adequate energy supply and cell membrane construction. These processes require cooperation between multiple enzymes. Increased reliance on oxidative phosphorylation (OXPHOS) has been reported as a distinctive hallmark of chemotherapy resistance cancer cells in numerous tumor types (4, 5). The unique peritoneal metastasis microenvironment of ovarian cancer is composed of tumor cells, immune cells including CD4^+^ and CD8^+^ T cells, tumor-associated macrophages (TAMs), natural killer cells (NK cells), dentritic cells (DCs), and myeloid-derived suppressor cells (MDSCs), as well as stromal cells including adipocytes, fibroblasts, human peritoneal mesothelial cells, and endothelial cells. The crosstalk between stromal cells, immune cells, and cancer cells enhances proliferative and drug-resistant phenotypes of cancer cells through the exchange of cytokines and activation of multiple downstream signaling pathways. Ovarian cancer cells also form a nutritional coupling relationship with stromal cells in TME, characterized by mutual metabolic dependency, particularly in terms of nutrient uptake, utilization, and sharing, indicating that these metabolic interactions create a cooperative “ecosystem” where tumor cells and surrounding cells rewire the metabolism pathways, supporting cancer growth and evading immune attack. Until recently, the stromal TME contribution to this metabolism reprogramming has not been fully appreciated, particularly in ovarian cancer.

These notable changes in metabolic pathways act as stress responses, allowing cancer cells to adapt to harsh TME, leading to chemotherapy resistance. Here, we summarized the detailed mechanisms of altered lipid metabolism influencing the immune, stromal and ovarian cancer cells in TME, gaining a better understanding of carcinogenesis, metastasis, and chemoresistance occurrence, providing novel insights into chemotherapeutic strategies, and finding solutions for reversing the immunosuppressive TME.

## 2 Lipid metabolism involved processes

### 2.1 Lipid uptake

**Figure 1:**
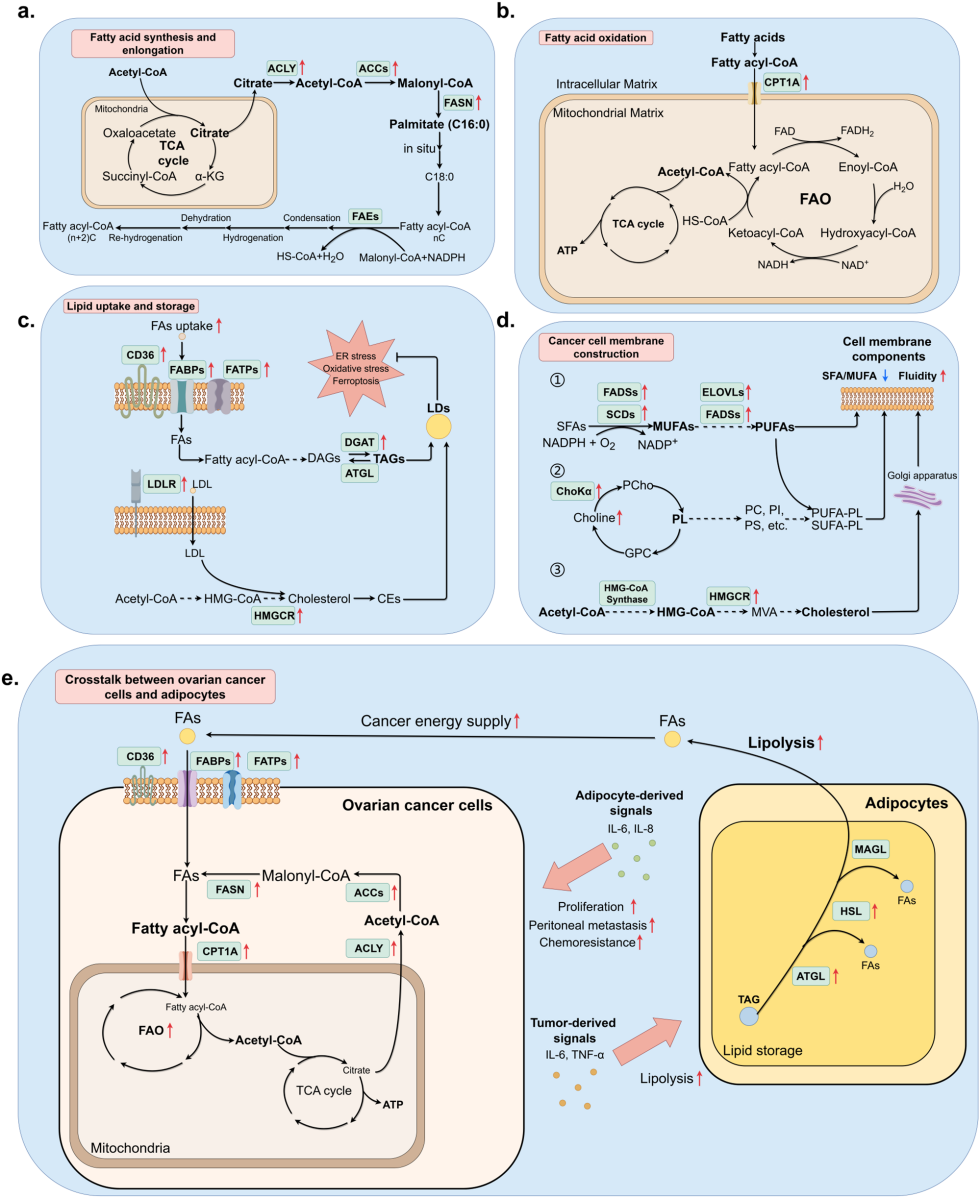
Lipid metabolic pathway alteration and crosstalk between ovarian cancer cells and adipocytes. (By Figdraw.) **(a)** Endogenous FAs *de novo* synthesis was elevated in ovarian cancer cells under the demand of original materials for higher FAO rate, membrane structure construction, and forming signaling factors, which facilitates tumor progression. **(b)** FAO correlated process was accelerated due to the over-expression of CPT1A, which is responsible for transporting fatty acyl-CoA from cytosol to mitochondrial matrix. **(c)** Lipids are first processed into storage form before being accumulated and stored in LDs. Correlating enzymes that turn FAs into TAGs have been found up-regulated in cancer cells, which is consistent with the fact that high LD accumulation is a type of stress response. **(d)** Cell membrane components consist of phospholipids, polyunsaturated FAs, and cholesterol to maintain the integrity and fluidity of membranes, which often relates to adhesion or metastasis of malignancies. **(e)** The crosstalk between ovarian cancer and adipocytes involves the secretion of pro-tumor factors and increased lipolysis of adipocytes, providing ovarian cancer cells with more FAs for FAO.

Many cancers (colon, breast, prostate, lung, ovarian cancer, and hematologic malignancies) stimulate lipolysis in adipocytes, followed by the uptake of FAs from the surrounding adipose tissue. The FAs enter the cancer cell through specific FA receptors and binding proteins (e.g., CD36, FATP1) and are used for membrane synthesis, energy metabolism, or lipid-derived cell signaling molecules (derivatives of arachidonic and linolenic acid). The access to increased lipids in ovarian cancer relies on both endogenous and exogenous pathways. Endogenous pathway depends on the intracellular *de novo* lipid synthesis, which utilizes acetyl-CoA as substrate. Exogenous pathway of lipid uptake requires the assistance of several lipid transporters, namely CD36, FA-transport proteins (FATPs), as well as FA-binding proteins (FABPs) (**Figure 1c**). The over-expression of CD36, FATPs, and FABPs had been indicated in ovarian cancer and other malignancies, correlating to cancer proliferation and aggressive behavior including metastasis (6). CD36 is a transmembrane glycoprotein receptor with a high affinity for long-chain FAs and cholesterol facilitating transmembrane passage and mediating intracellular trafficking via FABPs and endosomes. CD36-mediated activation of SRC/MAPK, AKT/GSK3β/β-catenin signaling axes, STAT3, and SOX2 had been shown to induce epithelial-mesenchymal transition (EMT) and promote proliferation, cancer stemness, metastasis as well as drug resistance (7). FABPs have been shown to directly promote ovarian cancer metastasis by facilitating the transcellular delivery of FA from omental host adipocytes to the ovarian cancer cells (8). Recent studies also indicated the inhibition of FATPs sensitizes ovarian cancers to oncolytic virus therapy via lipid modulation of the TME (9). Taken together, these studies illustrate that ovarian cancer promotes lipolysis and FAs uptake through transporters including CD36, FATPs, and FABPs, fueling tumor growth, metastasis, and drug resistance. Targeting these lipid metabolic pathways could enhance therapeutic responses.

### 2.2 *De novo* fatty acid synthesis

The *de novo* FAs synthesis is over-expressed in human ovarian cancer and in most common human solid tumors. The *de novo* FAs synthesis is mostly based on two key rate-limiting enzymes, namely acetyl-CoA carboxylase 1 (ACC1) and FA synthase (FASN). Before the biological synthesis of lipids begins, citrate obtained from the tricarboxylic acid cycle (TCA cycle) was transported into the cellular matrix and catalyzed to acetyl-CoA by ATP-citrate lyase (ACLY). ACCs can start the synthesis process via carboxylating acetyl-CoA to malonyl-CoA, while FASN continues catalyzing both acetyl-CoA and malonyl-CoA to form saturated FAs (SFAs) palmitate (C16:0), which are then provided for FAs and cholesterol synthesis. Extension of 16-carbon palmitate requires the involvement of FAs elongases (FAEs), the process is completed in the endoplasmic reticulum (ER) or mitochondrion, with NADPH as the hydrogen provider. After condensation, hydrogenation, dehydration, and rehydrogenation, two carbons are added. Normally the reaction in ER can extend palmitate to 24-carbon FA, whereas the reaction in mitochondrion can extend to 24 to 26-carbon FAs (**Figure 1b**).

ACLY catalyzes the citrate produced from the TCA cycle, either from glucose by glycolysis or glutamine, thus ACLY is considered a bridge connecting glycometabolism and lipid metabolism. ACLY is transcriptionally regulated by sterol regulatory binding element binding protein 1 (SREBP1), yet other factors such as insulin, glucagon, and TGF-β can also enhance the phosphorylation of ACLY (10). In ovarian cancer, the expression of ACLY is observed to have elevated compared to normal ovarian tissues, and increased expression level of phosphorylated ACLY in ovarian cancer was commonly associated with cancer grade, FIGO stage, and poorer prognosis. Pharmaceutical blockade of ACLY was reported to reverse the acquired cisplatin resistance in ovarian cancer (11).

FASN facilitates the rapid proliferation of tumors through the synthesis of palmitic acid (80%), stearic acid (10%), and myristic acid (10%). The accumulation of SFAs alters membrane physical properties by reducing fluidity and permeability, which correlates to higher risks of cancer progression (12, 13). The activity or expression of FASN is recognized and regulated by SREBPs through the binding site of its proximal promoter. The SREBP-FASN axis can be regulated by the PI3K-AKT signaling pathway and many proteins, including membrane-bound transcription factor protease site 2 (MBTPS2), CD36, and spindle protein 1 (SPIN1). Pharmacological inhibition of FASN using orlistat, C75, and TVB-2640 had achieved significant effects in ovarian cancer treatment as well as reversing chemotherapy resistance. The changes in FASN activity are considered the stress response of cancer cells reacting to changes in TMEs and have been identified as a significant contributor to the proliferation, metastasis, and progression of cancer.

The role of ACC1 in tumor progression, metastasis, and response to treatment is regulated by protein phosphorylation, allosteric modulator binding, and protein-protein interactions. AMP-dependent protein kinase (AMPK) phosphorylates ACCs, thus inactivating the enzyme, whereas protein phosphatase 2A dephosphorylates ACCs, activating the enzyme to produce malonyl-CoA. An ACC allosteric inhibitor, 5-tetradecepoxy-2-furanoic acid (TOFA), regulates ovarian cancer proliferation and cell cycle progression, ACC1 has been regarded as an attractive therapeutic target for ovarian cancer. Therefore, *de novo* FAs synthesis is upregulated in ovarian cancer, driven by increased ACLY, ACC, and FASN-mediated enzymatic activity, which promotes proliferation, metastasis, and chemoresistance.

### 2.3 Fatty acid oxidation

The increase of FAO, or β-oxidation, had long been noticed as a cancer hallmark, often correlated to the enhancement of FAs storage (**Figure 1a**). FAO is the major source of energy supply in cancer cells, meeting the requirement for high energy consumption during rapid cell division. Carnitine palmitoyltransferase 1A (CPT1A) is the rate-limiting enzyme in FAO, mediating the transportation of FAs from the intracellular matrix to the mitochondrial matrix, where FAs are oxidized to produce acetyl-CoA as an essential source for ATP, NADH, and FADH2 production in the TCA cycle. In our previous research, CPT1A had been identified to be differentially expressed with significance in paclitaxel-resistant ovarian cancer cell line A2780, and inhibition of CPT1A resulted in a prominent decrease of paclitaxel-resistant phenotype (14). Other studies revealed CPT1A as a clinical biomarker of platinum resistance using multiomic analysis (15). Ovarian cancer cells elevate the expression of CPT1A to maintain a high FAO level. CPT1A interacts with many cellular signaling pathways, including c-MYC and AMPK in breast cancer. The target also promotes cancer proliferation, metastasis, or therapeutic resistance through oncogenic signaling pathways including PI3K/AKT/mTOR, VEGF, ERK, and Src pathways. Furthermore, over-expression of CPT1A can also promote EMT and cancer cell stemness, leading to the invasion and metastasis of cancer cells (16). Thus, inhibiting FAO is an attractive means for ovarian cancer, given its critical role in sustaining tumor aggressiveness and treatment resistance.

### 2.4 Lipid storage

Lipid storage in cancer cells is mainly completed via the accumulation of LDs. Cancer cells introduce LDs as powerful methods to ensure energy supply and intracellular redox balance, modulate autophagy and mediate cellular membrane biosynthesis, and protect cancer cells from damage including ER stress, ferroptosis, and lipid peroxidation, therefore promoting cancer proliferation. Different types of lipids have corresponding storage forms. Emerging research indicates that LDs promote the proliferation, migration, and survival of cancer cells by alleviating cell stress and/or providing substrates for membrane lipid synthesis and FAO (17). Fatty acyl-CoA, the derivative of FAs, was converted into diacylglycerols (DAGs), further catalyzed into triacylglycerols (TAGs) by diacylglycerol acyltransferases (DGATs), and stored in LDs in the form of TAGs, whereas exogenous and endogenous cholesterols and other sterols are stored in the form of sterol esters such as cholesterol esters (CEs) (**Figure 1c**). LDs accumulation is associated with poor clinical prognosis, LDs marker adipophilin may serve as an independent indicator of a poor prognosis in ovarian cancer. PFK158 downregulates PLA2G3 (Group III Phospholipase A2) in ovarian cancer cells and human-derived primary ascites cells, inhibits LD biogenesis, decreases cell growth, and sensitizes the cells to platinum drug-mediated cytotoxicity (18). Tirinato et al. discovered that an increased number of LDs is a characteristic of radioresistant cancer cells in breast, bladder, lung, glioma, and prostate cancers. Restoring LD levels makes cancer cells more radiosensitive and enhances the efficacy of radiotherapy (19). Together, these data suggest that DGATs, PLA2G3 and possibly hormone-sensitive lipase could be promising targets for anticancer treatment. Therapies targeting LD biogenesis, growth, and degradation might be promising avenues for treating cancer. However, further work is needed to validate these therapeutic targets and strategies.

### 2.5 Cancer cell membrane construction

The biosynthesis of cell membranes relies on FA storage, which is the primary component. High fluidity and integrity of cell membrane is often correlated with metastatic behavior, this characteristic is based on the changes in membrane lipid composition, especially monounsaturated FAs (MUFAs) and polyunsaturated FAs (PUFAs) increase. This process is catalyzed by stearoyl-CoA desaturases (SCDs), elongation of very-long-chain FAs gene family (ELOVLs), and FA desaturases (FADSs). SCDs catalyze SFAs into MUFAs, including palmitoleic acid (C16:1) and oleic acid (C18:1), whereas ELOVLs and FADSs conduct the conversion between PUFAs (**Figure 1d**) (20). Previous research proved that elevation of SCDs was observed in multiple malignancies and often correlates to tumorigenesis, the homeostasis of the SFA/MUFA ratio is mediated by SCD1, which is an important cancer risk assessment factor; the conversion between SFA and MUFA relates closely to cancer prognosis. Pharmaceutical inhibition and genetic ablation of SCD1/FADS2 retarded tumor growth, cancer stem cell (CSC) formation, and reduced platinum resistance in ovarian cancer (21). PI3K/AKT/mTOR signaling pathway activates SREBP1, further modulating SCD1 for higher MUFA biosynthesis and protecting cancer cells from lipid peroxidation and ferroptosis (22). Inhibition of SCD1 in ovarian cancer enhances the sensitivity to ferroptosis inducers (23). Study conducted by Wang et al. pointed out that adequate PUFAs in omental conditioned medium or ascites suppressed RhoA-GTPase activities, further downregulated nuclear YAP1 in MФs, leading to increased protumoral M2-type TAM polarization accompanied by elevated OXPHOS metabolism. Loss of YAP1 had also been reported in ovarian cancer metastatic tissues in the same research, suggesting clinical relevance (24). Other component shifts in cancer cell membranes include increased phospholipids and cholesterol. Members of phospholipids, particularly phosphatidylcholine, contribute to most parts of the cellular membranes and produce lipid second messengers, promoting metastasis and chemotherapy resistance. As the key enzyme of phospholipid metabolism, Choline kinase (ChoK) has been reported to be activated in numerous types of cancer, and overexpression of ChoKα contributes to ovarian cancer progression, metastasis, and aggressiveness (25). Thus, Cancer cell membrane composition, particularly elevated FAs (MUFAs/PUFAs) mediated by SCDs, ELOVLs, and FADSs, promotes metastasis and therapy resistance. Targeting these pathways may suppress tumor growth and sensitize cells to therapy.

Cholesterol is another factor for maintaining membrane integrity and fluidity, which is endogenously synthesized via the mevalonate pathway utilizing acetyl-CoA as the starting material, further involving the biosynthesis of 3-hydroxy-3-methylglutaryl-CoA (HMG-CoA), mevalonic acid (MVA), and squalene, as well as conversion into other molecules, finally catalyzed into cholesterol. Synthesized cholesterol in the ER is transported to the cell membrane through the Golgi apparatus. Similar to other lipid metabolism key enzymes, SREBPs are the transcription factors of cholesterol synthesis rate-limiting enzyme HMG-CoA reductase (HMGCR), especially SREBP2. Excess cholesterol is removed from the cell via efflux mediated by ATP-binding cassette transporter A1 (ABCA1) (26). The reprogramming of cholesterol metabolism in both cancer cells and stromal TME can promote tumor growth, migration, and angiogenesis. Therefore, inhibiting cholesterol metabolism pathways is likely to substantially improve cancer treatment.

## 3 Lipid metabolism alteration contributes to immunosuppressive TME through crosstalk between stromal cells and immune cells.

### 3.1 Stromal cells

#### 3.1.1 Cancer-associated adipocytes

Ovarian cancer represents a type of cancer that is associated with adipocyte-rich, highly hypoxic microenvironments. Since peritoneal metastasis is a characteristic feature of ovarian cancer, this may suggests that ovarian cancer cells prefer a lipid rich microenvironment (27, 28). In recent decades, cancer-associated adipocytes (CAAs) aroused interest in cancer chemoresistance research (**Table 1**). Most studies choose ovarian cancer to correlate obesity and drug-resistance responses since ovarian cancer is one of the most obesity-associated types of cancer.

**Table 1:** Stromal cells in the TME.

Stromal cells	Lipid metabolism alteration	Factors secretion	Cancer types	Tumor proliferative effects	Reference
Cancer-associated adipocytes	Lipolysis↑ Lipid uptake↑ CD36 expression↑	LEP, MCP-1, TIMP-1, miRNA, HGF, FGF, IL-6, IL-8, IL-33	Ovarian cancer	Therapeutic resistance, Metabolic reprogramming, Increase metastatic capability	(40)
Cancer-associated fibroblasts	CD36 expression↑ Lipid Uptake↑ Lipogenesis↑	VEGF, TGF-β, IL-6, uPA, COX-2, CXCL1, CXCL10, CCL5,CRMP2	Ovarian cancer Colorectal cancer	Promote tumor growth, Increase metastatic capability, Extracellular matrix remodeling Generate alternative carbon sources for cancer cells	(41, 42)
Cancer-associated mesenchymal stem cells	FASN expression↑ FAS↑	SDF-1, CXCL1, CCL2, IL-8, CCL2, ITLN1	Ovarian cancer Oral squamous cell carcinoma	Promote tumor growth, Enhance chemotherapy resistance, Increase the cancer stem cell-like (CSC) pool, Increase tumor-associated fibrosis, Angiogenesis	(43, 44)
Mesothelial cells	LCN2 expression↑ Increase ovarian cancer lipid transport and accumulation	LPA, IL-6, IL-8, SDF-1, CX3CL1, CCL2, ITLN1	Ovarian cancer	Promote tumor growth, Increase adhension and metastatic capability	(45)
Endothelial cells	Modulate glycerophospholipids metabolism of ovarian cancer cells	IL-8, VEGF	Hepatocellular carcinoma Ovarian cancer	Tumor angiogenesis Tumor cell proliferation and invasion	(46, 47)

CAAs directly contribute to ovarian cancer progression mostly via increased lipolysis and produce more FAs, which are further transferred to ovarian cancer cells and enter through CD36, FATPs, and FABPs, providing energy for rapid cellular division and metastasis of ovarian cancer cells through enhanced FAO. The adequate lipids from adipocytes significantly support tumorigenesis and stemness of cancer cells. CAAs also secrete multiple cancer-associated adipokines that function in extracellular matrix remodeling, metastasis, chemotherapy resistance, and EMT promotion, such as leptin, monocyte chemoattractant protein-1 (MCP-1), tissue inhibitor of metalloproteinase-1 (TIMP-1), adiponectin, exosomal microRNAs, plasminogen activator inhibitor 1 (PAI-1), tumor necrosis factor-alpha (TNF-α), hepatocyte growth factor (HGF), fibroblast growth factor (FGF), interleukin-6 (IL-6), IL-8, and IL-33 (**Figure 1e**). Omental adipocytes undergo pyroptosis upon exposure to IL-6 and IL-8 produced by ovarian cancer cells, triggering the release of ATP that enhances macrophage infiltration and free FAs which are taken up by ovarian cells, thereby contributing to increased chemotherapy resistance. Though CAAs can modulate ovarian cancer metastasis, studies also reported that ovarian cancer can induce CAA formation via activating the TGF-β1/SMAD3/TRIB3 pathway in reverse, which suppresses the phosphorylation of CEBPβ. Then, CAAs secrete collagen I, collagen VI, and fibronectin to remodel the extracellular matrix and promote the adhesion of ovarian cancer cells (29).

Cancer cells mostly acquire chemoresistance via several mechanisms, such as increasing drug efflux, concealing molecular targets for drugs, intracellular inactivation of chemotherapies, dormancy maintenance, DNA damage repairing, survival signals enhancement, and self-regeneration. Several mechanisms have been proposed to explain chemoresistance influenced by CAAs. Chen et al. also revealed that the adipocyte-rich microenvironment promoted cisplatin and paclitaxel resistance in *in vitro* assays after applying human adipose tissue extracts. This chemoresistance phenotype is enhanced via upregulation of PPAR-γ/ABCG2 axis. Chemotherapy sensitivity can be restored after PPAR-γ knockdown using short hairpin RNA both in ovarian cancer cell lines and in nude mice models. The expression of PPARγ/ABCG2 was correlated to chemoresistance in ovarian cancer clinical specimens as well (30). Glycosylated angiopoietin−like 4 (ANGPTL4) secreted by CAAs could bind integrin α5β1 located on the surface of ovarian cancer cells, further activating down-stream c−myc/NF−κB pathway and stimulate the expression of the anti-apoptotic protein Bcl−xL, elevating the expression levels ABC family members such as ABCB1, ABCC1 and ABCG2 (31). Other views agree that extracellular vesicles (EVs) in the tumor micro-environment involve ovarian cancer chemoresistance, metastasis, and immune evasion mostly by transporting multiple miRNA EVs to facilitate CAA-ovarian cancer interactions.

Therefore, these researches demonstrate that CAAs promote tumor progression by releasing FAs (via CD36/FABPs) to fuel ovarian cancer cell growth, metastasis, and chemoresistance through enhanced FAO. Targeting the metabolic crosstalk between CAAs and tumors holds significant clinical promise for ovarian cancer treatment.

#### 3.1.2 Cancer-associated fibroblasts

Cancer‐associated fibroblasts (CAFs) are crucial factors in the TME that interact with cancer cells and promote carcinogenesis by secreting cytokines, chemokines, and EVs in ovarian cancer development (32). Single-cell transcriptomic profiling demonstrates that CAFs make up a heterogeneous population of cells with distinct functions. Cancer cells recruit and induce the transformation of the tumor-resident endothelial cells, epithelial cells, mesenchymal or hematopoietic stem cells, smooth muscle cells, and quiescent normal fibroblasts (NFs) to CAFs through paracrine signaling mechanisms (33). Activation of CAFs can be conducted via the JAK/STAT signaling pathway, the focal adhesion kinase (FAK) pathway, and the platelet-derived growth factor signaling (PDGF) pathway, as well as the NF-κB pathway (34). Several cytokines including IL-1β, IL-6, TGF-β also play pivotal roles in the activation process.

Compared to NFs, CAFs were observed to store more LDs, meanwhile showing microtubule organization centers amplification, inhibition of lipogenesis in CAFs inhibited lipid contents, and the number of microtubule organization centers. These evidences suggest that CAFs possess cancer-like phenotypes via lipid metabolism reprogramming and microtubule organization center amplification. A higher expression level of CD36 was observed notably in αSMA^+^/VIM^+^/PDGFRβ^+^ CAFs, which is associated with short-term survival. Considering CD36 plays a vital role in FA metabolism and immune regulation, its over-expression provides CAFs with FAs required as energy resources (35). Further, sphingolipid metabolism is also found to be altered in the stroma of ovarian cancer. Sphingosine kinases mediate the TGF-β signaling pathway via producing sphingosine-1-phosphate (S1P), which combines with S1P receptors, resulting in p38 MAPK phosphorylation. In this way, the tumor-promoting functions of CAFs were elevated.

CAFs are key contributors to tumor progression and therapeutic resistance through the remodeling of the tumor extracellular matrix composition and structure. Similar to modulation completed by CAAs, the crosstalk between cancer cells and CAFs depends on the communication conducted by EVs via receptor-ligand interactions or membrane fusion. The major component of the tumor extracellular matrix structure is fibrillar collagen (36). In brief, CAFs remodel extracellular matrix via stiffening collagen into short thick fibrils, up-regulating lysyl-oxidases (LOXs) and matrix metalloproteinases (MMPs), down-regulating hyaluronidases (HYALs), as well as secreting chemokines such as periostin, which changes tumor immune microenvironment by inducing migration of protumorigenic-M2 macrophages and correlates with lower tumor immune infiltration. In this way, CAFs form premetastatic niches essential for cancer cell invasion.

During chemotherapy, CAFs protect ovarian cancer cells in multiple ways. CAFs can activate several signaling pathways in ovarian cancer cells by releasing growth factors including miRNAs, which help cancer cells tolerate DNA damage via promoting proliferation and cell cycle entry, as well as suppressing cell apoptosis (37). CAFs also reduce ovarian cancer cells’ uptake of chemotherapeutic drugs via elevating the expression level of lipoma-preferred partner (LPP), which promotes tumor angiogenesis and enhanced tumor vessel leakiness (38). Moreover, CAFs promote the EMT transition of ovarian cancer cells, which increases the self-renewal ability and enhances the stemness of cancer cells. Apart from the previously described capabilities, CAFs can also alter the immune cell milieu by suppressing the activity of immune effector cells meanwhile recruiting immune suppressor cells, therefore allowing cancer cells to escape immune surveillance (39).

Thus, we discuss the current understanding of CAF-immune interactions, their effect on tumor progression and therapeutic response, and the possibility of exploiting CAF-immune interactions as potential targets for cancer therapy. CAFs have emerged as major promoters of immune evasion. Targeting CAF-ovarian cancer crosstalk may thus offer dual benefits in restoring antitumor immunity and sensitizing tumors to conventional therapies. CAFs display significant functional heterogeneity, with specific subsets serving as prognostic biomarkers in ovarian cancer.

### 3.2 Immune cells

**Figure 2:**
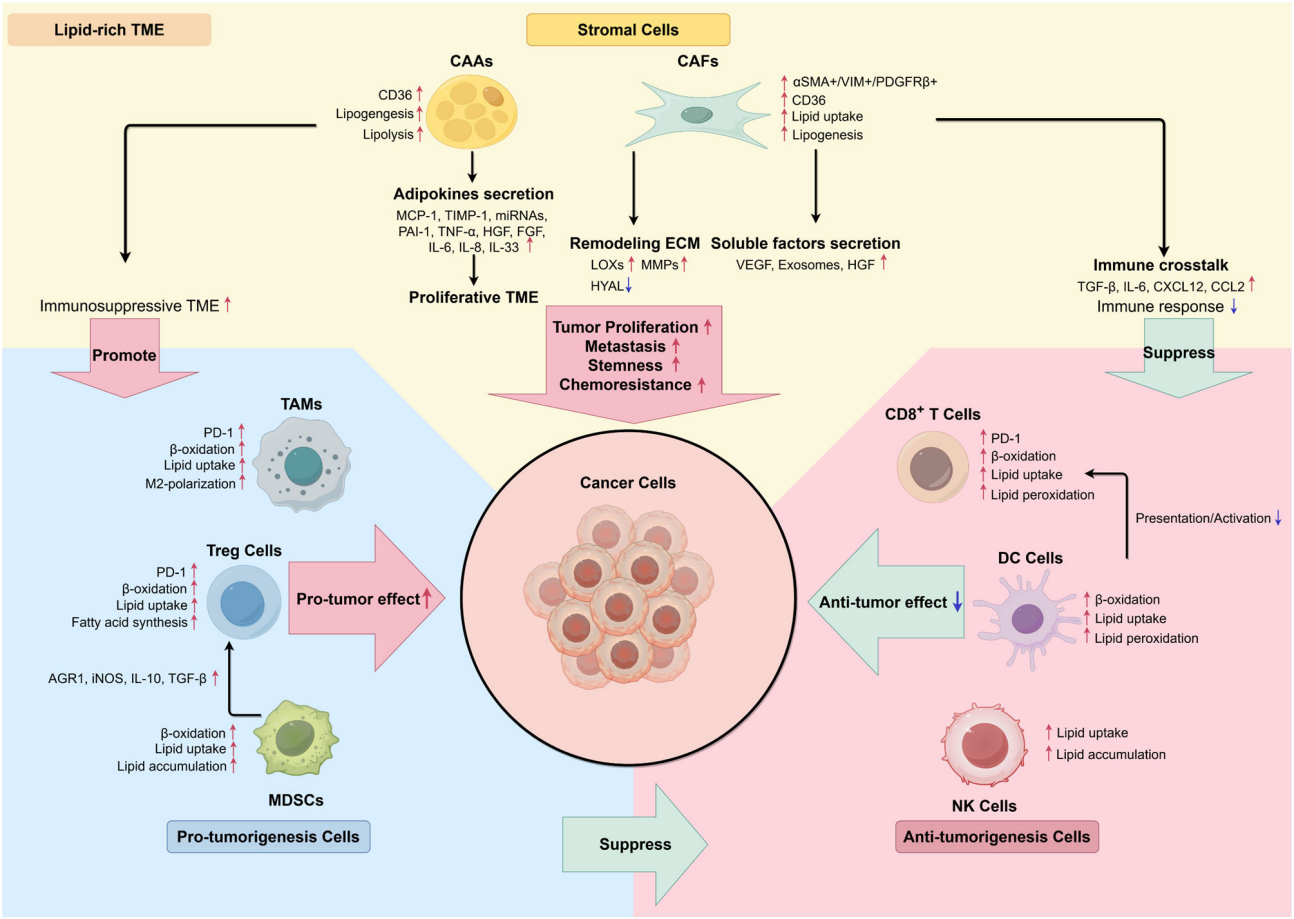
Interaction between stromal cells and immune cells in lipid-rich TME. (By Figdraw.) In the stromal TME, the interaction of cancerous, stromal, and immune cells tends to transform TME cells into a pro-tumor characteristic by secreting proliferative cytokines and lipid metabolites. Activation of lipolysis in adipocytes and adipokines secretion serve as energy resources and provide proliferative TME. Remodeling extracellular matrix completed by CAFs prepares cancer cells for metastasis, meanwhile inducing an immunosuppressive TME through secreting EVs and inhibiting CD8^+^ T cells’ immune response. Immune cells also exhibited enhancement in lipid metabolism, which results in immunosuppressive phenotypes, such as down-regulation of tumor-specific antigen presentation, increased anti-inflammatory signaling molecules, as well as increased M2-polarization of TAMs.

The TME refers to the cellular environment in which tumor cells exist, of which immune cells are critical components. The localization and function of immune cells in the TME are complex and have a profound impact on the clinical outcome of patients (**Table 2**). The crosstalk between immune cells and stromal cells has been extensively recognized in recent years (**Figure 2**).

**Table 2:** Immune cells in the TME.

Immune cells	Lipid metabolism alteration	Factors secretion	Cancer types	Function alteration	Reference
CD8^+^ T cells	FAO↑ Lipid uptake↑ Lipid peroxidation↑	IL-2, IFN-γ,TNF-α, CCL4, CCL5, TGFβ	Melanoma Colon cancer Ovarian cancer	Anti-tumor responses inhibition	(76, 77)
Natural killer cells	Lipid uptake↑ Lipid accumulation↑	IFN-γ, TNFα, IL-6, GM-CSF CCL5	Melanoma Chronic myeloid leukemia	Anti-tumor responses inhibition	(61)
Dendritic cells	FASN↑ Lipid uptake↑ Lipid peroxidation↑	IL-2, IL-10, IL-6, IL-12, CCR7, CCL2, CCL3, CCL4, TNF-α, MCP-1, CXCL10, IFN	Lymphoma Colon cancer Ovarian cancer	Antigen presentation process limitation Anti-tumor interferon responses inhibition	(78, 79)
Regulatory T cells	FAO↑ FAS↑ Lipid uptake↑ FASN↑	IL10, CTLA-4, IFN-γ	Melanoma Colon cancer	Inhibit CD8^+^ T cell activation Promote tumor growth	(55)
Tumor associated macrophages	FAO↑ Lipid uptake↑	TNFα, IL-1, IL-6, IL-12, IFN-γ	Ovarian cancer	Increase metastatic capability Promote angiogenesis Extracellular matrix remodeling Immune response inhibition M2-polarization	(80, 81)
Myeloid-derived suppressor cells	FAO↑ Lipid uptake↑ Lipid accumulation↑	MMP9, iNOS, ROS, VEGF, ARG1, TNFα, TGFβ, PGE2, IL-6, CCL2, CXCL1, CXCL2, CXCL5.	Fibrosarcoma Melanoma Lung cancer	Promote angiogenesis Increase metastatic capability	(82, 83)

#### 3.2.1 T cells

T cell function and fate are shaped by nutrient availability and the precise regulation of metabolic pathways in immunosuppressive malignancy. CD4^+^ T cells mainly include T helper cells (Th cells) and immune regulatory T cells (Tregs). CD8^+^ T cells mainly include cytotoxic T lymphocytes (CTL), they can secrete cytotoxic effector cytokines such as interferon-γ (IFN-γ), TNF-α, IL-2, IL-4, and IL-5. For effector and memory CD8^+^ T cells, the optimal uptake, trafficking, and catabolism of extracellular FAs are essential. CD8^+^ T cells that infiltrate ovarian tumors are retained in a dysfunctional state characterized by bioenergetic anomalies, aberrant activation of cellular stress responses, and negligible effector function that cannot be reversed through classical immunotherapeutic approaches (48, 49). In lipid-rich TME, CD8^+^ T cells take up more free FAs via CD36. The increased intracellular lipid content results in heightened lipid peroxidation and drives dysfunction in effector CD8^+^ T cells, leading to their exhaustion (50, 51). Lipid metabolism alteration impairs cytotoxic effects of T cells in most cases, the alteration may also act in contrast under certain situations. Hwang et al. noticed that overexpression of TAGLN2 after applying *TAGLN2* mRNA in ER-stressed CD8^+^ T cells increased their lipid uptake, mitochondrial respiration, and cytotoxic capacity. The contradiction suggested a potential clinical strategy of activating certain targets in ovarian cancer, therefore reshaping biological behaviors of CD8^+^ T cells in lipid-rich TME, enhancing their anti-tumor capability (52).

Current studies point out the importance of lipid metabolism in adaptive immune response. High levels of cholesterol in TME caused by tumor cells can elevate the expression levels of suppressive immune checkpoints of T cells, thereby making T cells lose the anti-tumor effect. Enhancement of FA metabolism also decreases the anti-tumor capability of CD8^+^ T cells. High expression of checkpoint targets including CTLA-4 and PD-1 in activated T cells inhibit glucose transporter 1 (GLUT1) and glycolysis, meanwhile increasing the expression of CPT1A and promoting cellular FAO level, accelerating the switch from T effector cells to T memory cells (53). The conversion between cell types reduces the quantity of anti-tumor T effector cells. Ma et al. (54) discovered the expression level of immune checkpoints positively correlated with total cholesterol content in tumor-infiltrating CD8^+^ T cells, T cells with the highest cholesterol content were the most exhausted population and had the highest apoptosis rate in both colon cancer and myeloma patient samples. They proposed that cholesterol accumulation in tumor-infiltrating CD8^+^ T cells may be the cause of their exhaustion phenotype and impair their function in TME.

In the TME, T cells and other stromal or immune cells engage in bidirectional communication via cytokine secretion, influencing immune function through the regulation of lipid metabolism. In addition to affecting cellular signaling, adipocytes also influence CD8^+^ T cell function through secreted metabolites, CAAs-secreted leptin shifts the metabolism of CD8^+^ T cells from glycolysis to FAO. As a result, immune escape and metastatic ability of cancer cells are promoted. Recruitment of Tregs in TME is also widely noticed as a method for tumor immune escape, Capable of autocrine immunosuppressive cytokines, Tregs secrete tumor growth factor-β (TGF-β), IL-10, and IL-35 to inhibit the proliferation and activation of T cells. The activity of SREBP and downstream FASN was found to be up-regulated in tumor-associated Tregs, which contributed to Tregs functional maturation, further correlated with tumor growth and anti-PD-1 immunotherapy inhibition (55). Moreover, Tregs are capable of promoting the SREBP1-dependent metabolic fitness of M2-type TAMs via repression of CD8^+^ T cell derived IFN-γ (56). Taken together, dysfunction of lipid metabolism undermines the regular function of stromal cells and differentiated T cells in TME, down-regulating immune response.

In summary, CD36-mediated lipid uptake in tumor-infiltrating CD8^+^ T cells typically induces ferroptosis and exhaustion through lipid peroxidation, targeted metabolic reprogramming can paradoxically enhance their mitochondrial fitness and cytotoxicity, revealing therapeutic opportunities to reverse T cell dysfunction in lipid-rich ovarian cancer microenvironments.

#### 3.2.2 NK cells

Ovarian cancer progression is associated with compromised immunosurveillance and is partly attributed to damage caused by the abnormal lipid metabolism of natural killer (NK) cells. Alteration of arachidonic acid metabolism impairs the cytokine signaling of NK cells through inhibition of STAT1 phosphorylation, as well as upregulating detoxification enzymes via induction of reactive oxygen species (ROS) (57). Encapsulating docosahexaenoic acid (DHA) and IL-15/IL-15Rα-secreting bioengineered adipocytes reactivate NK/CD8^+^ T cells in ovarian and colon cancer ascites, thus enhancing NK/CD8^+^ T cells anti-tumor activities (58).

Though NK cells have been extensively investigated in other cancer types, the influence of NK cells in ovarian cancer TME is poorly understood. Kobayashi et al. (59) applied transcriptional profiling of NK cells from Em-myc lymphoma samples, indicating the up-regulation of CD36, FABP4, FABP5, and PPARγ along with elevated FA level, proving NK cells in a lymphoma environment rewired lipid metabolism pathway at a substantial transcriptional level. This lipid metabolic reprogramming caused a suppressed production of IFN-γ and GZMB in human NK cells, impaired NK cell function. Besides, Tang et al. discovered that mTORC1/SREBP2 conducted abnormal cholesterol metabolism impairs antitumor immunosurveillance by causing NK cell dysfunction in hepatocellular carcinoma that develops from non-alcoholic fatty liver disease, inhibition of the mTORC1/SREBP2 may alleviate NK cell suppression to prevent obesity-promoted hepatocellular carcinoma (60, 61). Lehmann et al. also reported nerve growth factor receptor (NGFR) driving the up-regulation of SCD1, high production of unsaturated FA increased melanoma cell membrane fluidity and blocked the expression of NK cell-activating surface ligands such as CD112. Therefore, melanoma cells were able to escape from NK cell surveillance (62). Though lipid metabolic reprogramming mostly leads to NK cell suppressive TME, alteration in adipocytes may reverse the situation and bring novel clinical strategies. Zhang et al. applied IL-15-P2A-IL-15Rα-T2A-mCherry cDNA sequence stable transfected 3T3-F442A preadipocyte cells and encapsulated them with DHA. The bioengineered adipocytes lead specific expansion and activation of NK/CD8+ T cells response to the IL-15/IL-15Rα complex in malignant ascites, therefore reversing immunosuppressive phenotype of ascitic immune cells and enabling them to recognize and attack cancer cells (58).

These findings demonstrate that lipid metabolism critically regulates NK cell function in the TME, with dysregulated lipid processing, including excessive FAO, cholesterol accumulation, and impaired arachidonic acid metabolism. NK cell cytotoxicity is suppressed through inhibition of IFN-γ production, granzyme B secretion, and STAT1 signaling. Therapeutic strategies targeting these metabolic pathways could potentially restore NK cell function and enhance antitumor immunity. Bioengineered stromal cells also show significant therapeutic promise by restoring anti-tumor immunosurveillance through enhanced crosstalk with NK cells.

#### 3.2.3 Dendritic cells

DCs include classical DCs 1 (cDC1s), classical DCs 2 (cDC2s), plasmacytoid DCs (pDCs) and monocyte-derived DCs (moDCs). DCs have a strong antigen-presenting function that promotes adaptive immune responses and is important for mediating innate tumor immunosurveillance. Current immunotherapies include tumor vaccines, improving T lymphocyte function, application of immune checkpoint blockers, and adoptive cell therapy, all of which are initiated by the presentation of tumor-specialized antigens by antigen-presenting cells (APCs), especially DCs.

The abnormal accumulation of lipids in DCs is one of the major causes of DC dysfunction, this is mainly realized by up-regulating the expression levels of scavenger receptor A (SRA), lipoprotein lipase (LPL), and FABP4. Excessive lipid uptake is commonly seen as a sign of ER stress and oxidation damage because increased ROS mediates lipid peroxidation (63). The mechanism of lipid peroxidation in tumor-associated DCs to the ER stress response is mediated by the inositol-requiring protein 1 (IRE-1) and its downstream target X-box binding protein 1 (XBP1). This process of lipid accumulation in DCs is associated with reduced antigen processing ability, which is mainly caused by defective transportation of peptide-MHC (pMHC) class I complexes to the cell surface. This phenomenon further leads to the immune escape of tumors. Research conducted by Zhao et al. (64) revealed that melanoma-infiltrating DCs drove FAO over-activation via upregulating the expression of FA transporter proteins, thus inhibiting the activation of T cells and establishing immune-privileged sites. It was also demonstrated that high expression of FASN in ovarian cancer caused defective antigen presentation function of DCs, and consequently lower stimulatory effect for T cell proliferation. Therefore, lipid accumulation causes failure of DCs to induce an anti-tumor T-cell response, causing failure in immunotherapy.

Thus, lipid accumulation in DCs driven by elevated SRA, LPL, and FABP4 promotes ER stress through IRE1-XBP1 signaling and oxidative damage via ROS. These metabolic changes disrupt antigen processing by impairing pMHC-I transport, while excessive FAO and FASN overexpression further inhibit T cell activation. While these mechanisms are linked to immune evasion in melanoma and other cancers, their role in ovarian cancer remains poorly explored, highlighting the need for studies on metabolic interventions to improve DC function in this context.

#### 3.2.4 Macrophages

TAMs are usually divided into two types according to their polarization status: the M1-type TAMs and the M2-type TAMs. M1-type TAMs are induced by lipopolysaccharides (LPS) and IFN-γ, which possess pro-inflammatory and anti-tumorigenic phenotypes. The M2-type TAMs are induced by IL-4 and IL-10, which display both anti-inflammatory and pro-tumor functions.

Recent studies indicate TAMs in lipid-enriched TME accelerate ovarian cancer progression through lipid metabolic reprogramming. Adequate PUFAs and their derived metabolites in the TME of ovarian cancer mediate the crosstalk between ovarian cancer cells and TAMs, participating in the regulation of the signaling pathways of TAMs, affecting protumoral M2-type TAM polarization and functional characteristics, creating an immunosuppressive TME and eventually accelerating ovarian cancer progression and peritoneal metastases. TAMs promote membrane-cholesterol efflux and depletion of lipid rafts of ovarian cancer cells. Genetic deletion of ABC transporters, which conduct cholesterol efflux, prohibits the tumor-promoting functions of TAMs and reduces tumor progression. MCP-1 is the essential factor for TAM recruitment, the positive correlation between MCP-1 levels, TAMs, and tumor progression proposes that peritoneal metastasis of ovarian cancer is conducted by omental adipocytes which secrete MCP-1 and modulate the biological behavior of TAMs and ovarian cancer cells through MCP-1/CCR-2 axis. Apart from inducing M2-type TAM polarization, lipid metabolic alteration may directly influence TAMs dysfunction and ovarian cancer progression. Luo et al. revealed that PLIN2, a specific LD surface target, directly promoted lipid accumulation in ascites-associated macrophages, which further elevated the expression levels of SPP1 and CXCL8, and facilitated ovarian cancer progression and metastasis (65). Hence, TAM lipid metabolism and its influence on cancer progression and metastasis may offer new aspects for developing anti-tumor treatments targeting TAMs.

Increasing evidence indicates that lipid metabolism reprogramming, encompassing FA uptake and utilization, and cholesterol efflux, controls M2-type TAM polarization and further impacts the tumorigenesis of various cancers. FA uptake in TAMs is mainly conducted by the over-expression of CD36 and FABPs, TAMs uptake the tumor cell-derived FAs via CD36, especially monounsaturated long-chain FAs. PPAR-γ is a ligand-activated nuclear receptor, which can bind to the response element presented in the proximal region of the CD36 promoter to regulate the expression of CD36 and further promote the process of lipid uptake, meanwhile increasing lipid accumulation via FABP4/PPAR pathway, thus forming a positive feedback and promote M2-type TAM polarization, resulting in highly immunosuppressive TME (66).

Tumor cells mediate membrane cholesterol efflux in TAMs via secreting hyaluronic acid, causing lipid raft depletion and promoting STAT6 and PI3K-mTORC2-Akt signaling pathways activation, which enhanced IL-4 signaling and inhibited gene expression induced by IFN-γ, eventually induced TAMs toward the M2 phenotype.

The specific deletion of CPT2, which is located on the inner mitochondrial membrane that functions in FAO, was found to cause the impeding M2-type TAM polarization in mice models (67). The pro-tumor function of M2-type TAMs is extensively investigated, they promote tumor growth, metastasis, and tumor angiogenesis, meanwhile, it is been widely reported that M2-type TAMs promote therapy resistance, and removal of TAMs improves the efficacy of docetaxel in castration-resistant prostate cancer (68).

Therefore, in ovarian cancer’s lipid-rich microenvironment, M2 polarization is driven by PUFA metabolites, CD36/FABP4-mediated FA uptake, PPARγ activation, and cholesterol efflux via ABC transporters. These findings highlight lipid metabolism as a central regulator of TAM function in ovarian cancer, offering promising therapeutic targets to disrupt the pro-tumorigenic TME.

#### 3.2.5 Myeloid-derived suppressor cells

MDSCs are heterogeneous populations of immature myeloid cells which involved in tumor proliferation, metastasis, and immune tolerance. Typically, MDSCs impair anti-tumor immune responses by blocking the proliferation and antitumor activities of effector CD8^+^ T cells, multiple pathways involved in this process, including elevated expression of arginase 1 (Arg1), secreting immunosuppressive cytokines such as IL-10, inducible nitric oxide synthase (iNOS) and ROS production. MDSCs also support the *de novo* development of Treg cells through TGF-β-dependent and TGF-β-independent pathways (69). Further forming an immunosuppressive TME.

Lipid metabolism alteration plays a vital role in MDSCs functioning. Dong et al. indicated that tumoral NAC1 directly enhanced the transcription of CXCL16 by binding to CXCR6, hence promoting MDSCs recruitment to the tumor. Inhibition of NAC1 reduced the recruitment and immunosuppressive function of MDSCs in the TME, led to significant increases of cytotoxic tumor infiltrating CD8^+^ T cells, potentiated anti-PD-1 therapy, and suppressed tumor progression in ovarian cancer (70). Bioactive lipid prostaglandin E2 (PGE2), a derivative of arachidonic acid, can be produced by MDSCs and increases stem cell-like properties, as well as PD-L1 expression in epithelial ovarian cancer (71).PEG2 also regulates PD-L1 expression in MDSCs via COX2/mPGES1/PGE2 pathway, which reprograms PGE2 metabolic pathways in TME and provides an opportunity to reduce tumor immune suppression (72). Recent studies also conclude that tumor-associated MDSCs up-regulate FAO as a primary energy source, this is supported by higher mitochondrial mass, increased FA uptake via CD36 and FA transport protein 4 (FATP4), and over-expression of lipid metabolism key enzymes such as CPT1 and acyl-CoA dehydrogenase (73). Lipid accumulation in MDSCs enhances their immunosuppressive effect, primarily due to oxidized lipids produced by ROS and MPO. Polyunsaturated FAs, being highly prone to oxidation, facilitate MDSC-mediated suppression of effector CD8^+^ T cells in the TME. Xin et al. (74) identified the role of proto-oncogene PIM-1 in PPAR-mediated lipid metabolism in myeloid cells. They reported a strong relationship between PIM-1 expression, increased FAO, immune checkpoint blockade treatment resistance, and PD-L1 blockade, inhibiting PIM-1 showed reduced MDSCs population and improved the cytotoxic killing effects of CD8^+^ T cells. Thus, these studies indicate that excessive lipid uptake and oxidation lead to resistance against PD-1/PD-L1 pathway treatments (75). Pharmacologic inhibition targeting MDSCs lipid metabolic key enzymes may alleviate tumor development and improve the anti-tumor effects of clinical immunotherapy. These findings establish lipid metabolism as a promising therapeutic target to counteract MDSC mediated immunosuppression and enhance immunotherapy responses in ovarian cancer.

## 4 Conclusion

Lipid metabolic reprogramming in ovarian cancer cells is characterized by enhanced FAs and cholesterol uptake, FA synthesis, FAO, and lipid storage, provides energy supply, prepares raw materials for cellular membrane construction, and signaling factors formation, these biological processes have notable meaning in the occurrence of cancer development, aggressive behaviors, and chemoresistance. In lipid-rich TME, function alteration mainly involves stromal and immune cells including CAFs, Tregs, CD8^+^ T cells, and TAMs, the crosstalk between these cells forms premetastatic niche and immunosuppressive TME, leading to metastasis and tumor immune escape, eventually promoting proliferative phenotype of ovarian cancer. Thus, this review provides novel aspects of targeting proliferative features via inhibiting lipid metabolism reprogramming in ovarian cancer and decreasing the lipid accumulation in stromal TME, thereby restoring the sensitivity of both chemotherapy and immunotherapy.

As described previously, since peritoneum is a lipid-rich environment, it is easy to explain why peritoneal metastasis happens and further causes ovarian cancer development and poorer prognosis. Novel technologies including metabolomics, proteomics, single-cell RNA sequencing, and spatial transcriptomics can be utilized to detect the accurate metabolic alteration taking place in the stromal TME cells, revealing the detailed mechanisms and impacts of intercellular communication. Lipid metabolism reprogramming in ovarian cancer manifested as multi-level systemic dysfunction. In detail, it involves changes in key enzyme activities, abnormal up-stream and down-stream modulation in metabolic pathways, and overall disorder of lipid-carbohydrate-amino acid metabolic network. Existing drugs targeting lipid metabolism exhibited limited clinical efficacy is fundamentally due to two reasons. Traditional small molecule single target inhibitors are prone to off target effects, meanwhile compensatory regulation of the metabolic network leads to failure of monotherapy. The present situation urges for the development of synergistic, multi-target therapy. Notably, the metabolic dysfunction exists not only in tumor cells, but widely in stromal cells and immune cells in the TME. Based on the understanding, two emerging strategies, namely bioengineered matrix/immune cell therapy and nanocarrier targeted delivery system, have opened up new attempts for metabolic intervention therapy of ovarian cancer.

Our review may be able to provide novel insights into inhibiting metastasis in ovarian cancer and promoting therapeutic efficacy. Taken together, the investigation targeting stromal TME holds promise in improving outcomes for patients with late-stage or developed drug resistance, and provides patients with the hope for a cure.
